# Expression and Characterization of *Geobacillus stearothermophilus* SR74 Recombinant *α*-Amylase in *Pichia pastoris*

**DOI:** 10.1155/2015/529059

**Published:** 2015-05-18

**Authors:** Sivasangkary Gandhi, Abu Bakar Salleh, Raja Noor Zaliha Raja Abd Rahman, Thean Chor Leow, Siti Nurbaya Oslan

**Affiliations:** ^1^Institute of Bioscience, Universiti Putra Malaysia, 43400 Serdang, Selangor, Malaysia; ^2^Department of Biochemistry, Faculty of Biotechnology and Biomolecular Sciences, Universiti Putra Malaysia, 43400 Serdang, Selangor, Malaysia; ^3^Department of Microbiology, Faculty of Biotechnology and Biomolecular Sciences, Universiti Putra Malaysia, 43400 Serdang, Selangor, Malaysia; ^4^Department of Cell and Molecular Biology, Faculty of Biotechnology and Biomolecular Sciences, Universiti Putra Malaysia, 43400 Serdang, Selangor, Malaysia

## Abstract

*Geobacillus stearothermophilus* SR74 is a locally isolated thermophilic bacteria producing thermostable and thermoactive *α*-amylase. Increased production and commercialization of thermostable *α*-amylase strongly warrant the need of a suitable expression system. In this study, the gene encoding the thermostable *α*-amylase in *G. stearothermophilus* SR74 was amplified, sequenced, and subcloned into *P. pastoris* GS115 strain under the control of a methanol inducible promoter, alcohol oxidase (*AOX*). Methanol induced recombinant expression and secretion of the protein resulted in high levels of extracellular amylase production. YPTM medium supplemented with methanol (1% v/v) was the best medium and once optimized, the maximum recombinant *α*-amylase SR74 achieved in shake flask was 28.6 U mL^−1^ at 120 h after induction. The recombinant 59 kDa *α*-amylase SR74 was purified 1.9-fold using affinity chromatography with a product yield of 52.6% and a specific activity of 151.8 U mg^−1^. The optimum pH of *α*-amylase SR74 was 7.0 and the enzyme was stable between pH 6.0–8.0. The purified enzyme was thermostable and thermoactive, exhibiting maximum activity at 65°C with a half-life (*t*_1/2_) of 88 min at 60°C. In conclusion, thermostable *α*-amylase SR74 from *G. stearothermophilus* SR74 would be beneficial for industrial applications, especially in liquefying saccrification.

## 1. Introduction

Amylase is a starch degrading enzyme of biotechnological and industrial significance, which has received much attention due to its economic benefits and technological significance. Although short growth and metabolite production are essential parameters for choosing microorganisms as sources of enzymes, other predominating factors may dictate microorganisms as main enzyme source. The main reasons are that microorganisms being controlled physiologically and physicochemically, high product yield compared to plant and animal sources, easy recovery in downstream process, cost beneficial while processing, and so forth. Moreover, the substrates, raw materials, and production systems are cheap. Several developed countries of Europe and USA and Japan are well-known for their commercial production of enzymes using microorganisms [1]. Alpha amylase (*α*-1,4-d-glucan glucanohydrolase and endoamylase; EC 3.2.1.1) cleaves internal *α*-1,4-glycosidase bonds in the long chains of starch to produce glucose, maltose, or dextrin [2]. The substrate specificities of *α*-amylase are different and their stability to temperature, acidic pH, and half-life characteristics remains crucial in successfully exploiting this enzyme in starch-processing industries [3–5].

Among bacterial sources, several strains of* Bacillus* and* Geobacillus* have been exploited for thermostable *α*-amylase production in industries [6]. In the past decades due to its great demand and widespread use in industries, the indigenous production of *α*-amylases is escalating in many countries worldwide. Alpha-amylases are widely utilized in starch liquefaction, starch saccrification, detergents, baking industry, breweries, digestibility in animal feed, and fibre and cotton desizing [7]. Despite its use in starch processing industry and other related areas, currently the scope of *α*-amylase application has widened its range to other fields like, clinical, medical, and analytical chemistry as an arrival of new frontiers in biotechnology which ranked *α*-amylase as the first among the various extra cellular enzymes [8].

*Geobacillus* sp., (formerly* Bacillus*) is known to produce *α*-amylase that can hydrolyze raw starch granules. Colonies of* G. stearothermophilus* were reported to have high *α*-amylase activity, when the organism was used in batch experiments to produce *α*-amylase [9]. A species of* Geobacillus* was isolated from a hot spring near Slim river of Perak state in Malaysia, which produced a thermostable *α*-amylase. Expression systems like* Escherichia coli* and* Saccharomyces cerevisiae* are well known for their cloning and expression capabilities. Such expression systems are disadvantageous in commercial point of view where overexpressed proteins transform as inactive forms or as inclusion bodies inside, ultimately resulting in low recovery [10]. Moreover, the newly isolated thermostable and thermoactive *α*-amylase from* G. stearothermophilus* is yet to be characterized. All these scenarios clearly underscore the pressing need of a yeast expression system that is more suitable for cloning and expression of the protein of interest extracellularly by its secretory pathway.

Yeast expression systems like the* Pichia pastoris* have great demand for the increased production of industrially important heterologous enzymes and proteins.* P. pastoris* is a methylotrophic yeast which possess excellent biotechnological attributes which includes its high cell density, high efficiency, strictly regulated alcohol oxidase gene (*AOX1*) promoter, and extracellular release of the protein of interest is easily achieved with less difficulties than other expression systems [11–14].* P. pastoris* expression systems are generally regarded as safe [15] and are widely used for the expression of many heterologous foreign proteins with a high success rate [16]. Despite many advantages, none has attempted or reported the successful *α*-amylase production in* P. pastoris*.

Since natural thermophilic isolates like* Geobacillus stearothermophilus* are not considered suitable because of their low yield and high production costs, bioprocess optimization and suitable expression system like* P. pastoris* involving recombinant DNA technology will lead to commercialization and/or industrial application. This study highlights the expression and characterization of thermoactive and thermostable* G. stearothermophilus* SR74 isolated *α*-amylase in* P. pastoris*. PCR cloning and sequencing of the *α*-amylase gene were also investigated with a special emphasis on the overexpression and recovery. Also biochemical characterization of crude and purified *α*-amylase in terms of its stability to temperature and pH are also studied.

## 2. Materials and Methods

### 2.1. Chemical, Reagents, and Media

Expression plasmid pPICZ*α*B,* P. pastoris* strain GS115-*his4*, and* E. coli* TOP10 (F-*mcr*A Δ(*mrr*-*hsd*RMS-*mcr*BC) *φ*80*lac*ZΔM15 Δ*lac*Χ74* rec*A1* ara*D139 Δ(*ara-leu*) 7697* gal*U* gal*K* rps*L (StrR)* end*A1* nup*G *λ*-) (used for subcloning and plasmid propagation), zeocin were purchased from Invitrogen, USA. Growth media Luria Bertani (LB) medium was obtained from Difco Laboratories. Bovine serum albumin was obtained (Sigma, USA). BioLine Sdn Bhd supplied PCR primers were used for subcloning.* Taq* DNA polymerase was purchased from Fermentas, USA.

### 2.2. Strains, Plasmids, and Culture Media

Mature *α*-amylase gene from* G. stearothermophilus* SR74 was amplified from the recombinant plasmid pET-32b/*α*-amylase. Empty host was used as a control for this experiment.* E. coli* TOP10 (F-*mcr*A Δ(*mrr*-*hsd*RMS-*mcr*BC) *φ*80*lac*ZΔM15 Δ*lac*Χ74* rec*A1* ara*D139 Δ(*ara-leu*) 7697* gal*U* gal*K* rps*L (StrR)* end*A1* nup*G *λ*-) was used for subcloning and plasmid propagation.* E. coli* containing recombinant pET-32b/*α*-amylase was grown in 10 mL Luria Bertani (LB) broth supplemented with 25 *μ*g mL^−1^ of ampicillin in a 28 mL volume Universal bottle. The bacterium was grown at 37°C for 16–18 h in the orbital shaker of 200 rpm agitation for the plasmid extraction.* Pichia pastoris* of 5 mL cultures were grown overnight in Yeast Peptone Dextrose (YPD) medium which contained yeast extract (1% w/v), peptone (2% w/v), and dextrose (2% w/v) in 50 mL conical at 30°C. YPD agar plates (same composition as YPD as mentioned above supplemented with 1% agar) containing 100 *μ*g mL^−1^ zeocin were used for the selection of* P. pastoris* transformants. Selection of different media components in order to get a better growth of the cultures was obtained from previously described methods [17]. The growth medium used was buffered minimal glycerol yeast extract (BMGY) medium containing yeast extract (1% w/v), peptone (2% w/v), yeast nitrogen base (1.34% w/v), biotin (4 × 10^−5^% w/v), glycerol (1% v/v), and potassium phosphate buffer (100 mM, pH 6.0). Buffered minimal methanol yeast extract (BMMY) medium served as induction medium that contained yeast extract (1% w/v), peptone (2% w/v), yeast nitrogen base (1.34% w/v), biotin (4 × 10^−5^% w/v), methanol (0.5% v/v), potassium phosphate buffer (100 mM, pH 6.0), and glycerol (1% v/v) replaced with methanol (0.5% v/v). The experiment was conducted in triplicate.

### 2.3. DNA Amplification and Cloning

The gene encoding mature *α*-amylase SR74 (without signal peptide) was amplified by PCR using the recombinant plasmid of pET-32b/*α*-amylase SR74 as a template. Primers pairs for the gene encoding *α*-amylase SR74 were obtained from NCBI website (http://www.ncbi.nlm.nih.gov/) with gene accession number: FJ997644. The primers also incorporated restriction endonuclease sites, so that the PCR products could be cloned into the final vector. The gene encoding mature *α*-amylase SR74 was amplified using the forward primer 5′-CGCCCACGTGGCCGCACCGTTTAACGGCAC-3′ incorporating a* Pml*I site and the reverse primer 5′-TGGTTCTAGACAAGGCCATGCCACCAACCGTGG-3′ incorporating* Xba*I site (underlined nucleotides indicate the restriction endonuclease* Pml*I and* Xba*I sites). PCR was carried out using* Taq* DNA polymerase and the reaction conditions were mentioned in Table 1. The purified amplicons and expression vector pPICZ*α*B were digested with* Pml*I and* Xba*I. The insert and vector were ligated and cloned into* E. coli* TOP10 according to manufacturer's instructions provided in the EasySelectTM* Pichia* Expression kit inserts. The constructed recombinant plasmid pPICZ*α*B/*α*-amylase fusion gene in frame with native* Saccharomyces cerevisiaeα*-factor secretion and polyhistidine-tag (C-terminal) was confirmed by automated sequencer (Applied Biosystems, USA).

### 2.4. Transformation into* P. pastoris* Strain

Transformation of the gene of interest into* P. pastoris* GS115 strains was achieved by electroporation method. The electrocompetent cell was prepared according to EasySelect* Pichia *expression kit manual with minor modifications. The recombinant plasmid pPICZ*α*B/*α*-amylase was linearized by using* Sac*I restriction endonuclease prior to gene integration into the* P. pastoris* genome. Transformants were selected on YPDS agar plates containing yeast extract (1% w/v), peptone (2% w/v), dextrose (2% w/v), 1 M sorbitol, and agar (2% w/v) supplemented with 100 *μ*g mL^−1^ zeocin at 30°C. Single colony of* P. pastoris* grown on YPDS was inoculated into 10 mL YPD broth and incubated overnight at 30°C under shaking conditions at 250 rpm. Then, 500 *μ*L of the culture was inoculated/transferred to a 500 mL volume DURAN Erlenmeyer flasks with baffles (DURAN Produktions GmbH & Co. KG, Mainz, Germany) containing 100 mL of BMGY and incubated in a shaking incubator at 30°C for 24 h at 250 rpm. The cells were harvested and adjusted to OD_600 nm_ = 10 in 50 mL of BMMY medium. The culture was induced with methanol (0.5% v/v) for 48 h with a 24 h interval. One mL of the culture was harvested, centrifuged at 3000 ×g for 10 min at 4°C and the supernatant was subjected to *α*-amylase assay using the DNS method with minor modifications [18].

### 2.5. Effect of Different Media Composition

Recombinant (GS115/pPICZ*α*B/*α*-amylase) was evaluated in different media formulations by following the method described previously [17]. Briefly, 1 mL of the culture from YPD broth was inoculated into each shake flask containing 100 mL of growth media (BMG, MMG, BMGY, and YPTG) for 48 h. The composition of each medium was as follows: BMG—yeast nitrogen base (1.34% w/v), biotin (4 × 10^−5^% w/v), glycerol (1% v/v), and potassium phosphate buffer (100 mM, pH 6.0); MMG—yeast nitrogen base (1.34% w/v), biotin (4 × 10^−5^% w/v), glycerol (1% v/v), and distilled water (pH 6.0); BMGY—yeast extract (2% w/v), peptone (2% w/v), yeast nitrogen base (1.34% w/v), biotin (4 × 10^−5^% w/v), glycerol (1% v/v), and potassium phosphate buffer (100 mM, pH 6.0); and YPTG—yeast extract (1% w/v), peptone (2% w/v), biotin (4 × 10^−5^% w/v), tryptic soy broth (0.2% w/v), and glycerol (1% v/v). To ensure growth of transformation, the recombinant *α*-amylase SR74 in minimal media (MMG and BMG) was supplemented with histidine (0.004%). After 24 h of cultivation, growth media (BMG, MMG, BMGY, and YPTG) were replaced with induction media MMM, BMM, BMMY, and YPTM. The culture were resuspended into 50 mL of induction media containing methanol 0.5% (v/v) instead of glycerol to a final OD_600_ = 10. Cultures were incubated at 30°C for 48 h at 250 rpm and induced with methanol at 24 h interval. Cultures were harvested and the supernatants were stored at −20°C until further analysis on *α*-amylase assay.

### 2.6. Effect of Different Concentrations of Methanol

Recombinant (GS115/pPICZ*α*B/*α*-amylase) was grown in YPTM medium as described above with final OD_600 nm_ = 10. Different concentrations of methanol (0, 0.5, 1.0, 1.5, 2.0, 2.5, and 3% v/v) were used to induce the *α*-amylase expression. After 48 h of cultivation, the cells were harvested and the supernatant was assayed for *α*-amylase activity.

### 2.7. Effect of Induction Time on the Expression of Recombinant *α*-Amylase

The recombinant* P. pastoris* GS115 which secreted the highest activity of the *α*-amylase was grown in 100 mL of YPTG medium in a 500 mL baffle flask at 30°C at 250 rpm. Cells were harvested at room temperature at 3000 ×g for 5 min and suspended in 50 mL of YPTM medium (same composition as YPTG except that glycerol was substituted with 1% methanol). The culture was then grown at 30°C at 250 rpm and induced with methanol (1%) for every 24 h from 0–192 h. An aliquot of 3 mL culture was taken at an interval of every 24 h and the expression of *α*-amylase was determined.

### 2.8. Purification of Recombinant *α*-Amylase Protein

The *α*-amylase fused with polyhistidine tag from recombinant supernatant was purified using immobilized metal affinity chromatography (IMAC) on 5 mL HiTrap IMAC FF, fast flow column with AKTA purifier system (Amersham Biosciences, USA). The column was equilibrated with binding buffer (20 mM sodium phosphate, 500 mM NaCl, pH 7.4). Filtered *α*-amylase supernatant (20 mL) was loaded to the column and the column was washed with the binding buffer. The *α*-amylase was eluted with the elution buffer (20 mM sodium phosphate, 500 mM NaCl, and 500 mM imidazole, pH 7.4) using a linear gradient of imidazole ranging from 20 to 500 mM. Eluted fractions were collected and assayed for *α*-amylase and protein. The active fractions were pooled and the homogeneity of the enzyme was determined using SDS PAGE. Aliquots of the purified *α*-amylase (0.5 mg) in eppendorf tubes were stored at −20°C.

### 2.9. Determination of *α*-Amylase Activity and Protein Content

Amylase activity was determined by DNS method as described earlier [18]. Briefly, 0.5 mL enzyme solution and 0.5 mL starch (1% w/v) in 50 mM phosphate buffer (pH 7.0) were mixed and allowed to react for 30 min at 60°C. Same amount of DNS reagent was added to each tube and incubated at 100°C for 10 min in order to stop the reaction by inactivating the enzymes. Simultaneously, the DNA molecules react with the reducing sugars released by the amylase in the same reaction step. Tubes which received enzymes after incubation with boiled DNS reagent served as control tubes. The tubes were allowed to cool to room temperature and the absorbance of the reaction mixture was read at 540 nm in a UV-Vis spectrophotometer. Reducing sugar was determined by comparing the absorbance at 540 nm of the assay solution with a maltose standard curve. One unit (U) of the enzyme activity was defined as the rate of production of 1 *μ*M of reducing sugar (as maltose) from 1% soluble starch as substrate in 1 min at 60°C and pH 7.0. The assay was conducted in triplicates and the results were expressed as the mean of experiment reading. Protein estimation was carried out using BSA as standard [19].

### 2.10. SDS PAGE and Western Blotting

Sodium dodecyl sulfate polyacrylamide gel electrophoresis (SDS PAGE) was performed using 12% polyacrylamide gel according to the method described earlier [20]. The proteins from SDS PAGE gel were transferred to nitrocellulose membrane by electroblotting according to the manufacturer's instruction provided in NOVAGEN standard protocol of His Tag Monoclonal Antibody.

### 2.11. Characterization of *α*-Amylase

#### 2.11.1. Effect of pH on *α*-Amylase Activity and Stability

The optimum pH for *α*-amylase activity was measured at different pH ranging from pH 4.0 to pH 12.0 at 60°C for 30 min. *α*-Amylase enzyme (50 mM) was incubated in different buffers of varying pH, namely, sodium acetate buffer (pH 4.0–6.0), potassium phosphate buffer (pH 6.0–8.0), Tris-HCl buffer (pH 8.0–9.0), glycine-NaOH (pH 9.0–11.0), and Na_2_HPO_4_-NaOH (pH 11.0–12.0). The residual activity was assayed using starch (1% w/v) as substrate in 50 mM phosphate buffer, pH 7.0.

#### 2.11.2. Effect of Temperature on *α*-Amylase Activity and Thermostability

The effect of temperature on purified *α*-amylase was determined at various temperatures ranging from 40 to 80°C. Thermostability of the enzyme was determined by incubating the enzyme at 60°C, 65°C, and 70°C for 30 min and the residual activity was assayed as mentioned above in Section 2.11.1.

## 3. Results and Discussion

### 3.1. Cloning and Sequence Analysis of *α*-Amylase Gene into pPICZ*α*B

The ORF of the *α*-amylase SR74 gene (accession number: FJ997644) consisted of 549 amino acids. Thirty-four amino acids representing the signal peptide were eliminated (no contribution in structural gene of *α-amylase* SR74) and the remaining 515 amino acids which belonged to the mature *α*-amylase gene were used for further investigation.* E. coli* TOP10 are considered one of the most suitable competent cells noted for their high-efficiency cloning and plasmid propagation which allows stable replication of high-copy number plasmids.* E. coli* has been used as a host to allow replications and maintain the constructed plasmids, as there is no yeast origin of replication in* P. pastoris* vector plasmid. pPICZ*α*B-*α*-amylase SR74 recombinant plasmid was transformed into* E. coli* TOP10 and the transformants were formed on LB agar containing 25 *μ*g/mL of zeocin. Colony PCR results confirmed the presence and orientation of the *α*-amylase SR74 gene in the inducible pPICZ*α*B expression vector.* AOX*1 primers amplified a 550 bp fragment of the *α*-amylase SR74 gene which was 1545 bp in size and positive transformants showed a band size of ~2.1 kb. Double digestion of the recombinant plasmid using restriction enzymes* Pml*I and* Xba*I produced two DNA fragments and hence the pPICZ*α*B/*α*-amylase SR74 recombinant plasmid was confirmed. A DNA fragment of 3.6 kb was identified as the inducible vector (pPICZ*α*B), whereas *α*-amylase SR74 gene had a molecular weight of 1.5 kb.

### 3.2. Transformation into* Pichia pastoris* Strain GS115

**Figure 1:**
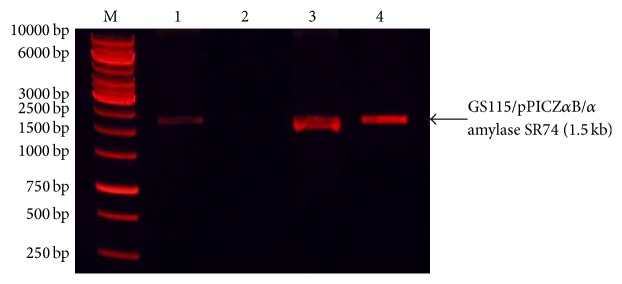
Gel electrophoresis of PCR products of three randomly selected* P. pastoris* GS115 transformant amplified using XbaI (forward) and PmlI (reverse) primers. M, 1 kb DNA ladder; Lanes 1, 3, and 4 are PCR amplicons of GS115/pPICZ*α*B/*α*-amylase transformants. Lane 2 has no band represents pPICZ*α*B expression vector.

Linearization of the vector by restriction digestion of 5′ to the* AOX*1 promoter (e.g., at the* Sac*I site of pPICZ*α*B) directs the integration of the plasmid to the homologous sites in the* P. pastoris* genome [21]. Integration at 5′*AOX*1 locus, using* Sac*I digestion, is an efficient straightforward way to generate recombinant clones for heterologous protein expression. Therefore, single digestion using* Sac*I restriction enzyme was performed on the selected plasmid which resulted in a linearized recombinant pPICZ*α*B/*α*-amylase SR74 DNA plasmid with a molecular weight of ~5.2 kb. Transformation of* P. pastoris* strains GS115 by electroporation method managed to observe the colonies growth. Integration of the expression cassette (5′ P_*AOX*1_, *α*-amylase gene, transcription terminator (TT) and zeocin) into the chromosome at a specific locus generated genetically stable transformants [22]. The four GS115/pPICZ*α*B/*α*-amylase SR74 transformants were randomly selected with zeocin resistant gene (*Sh ble*) as a selectable marker from the integration vector. The positive transformants of the PCR results showed the presence of the gene fragment of ~1.5 kb which confirmed the successful integration of *α*-amylase SR74 gene into the genome of* P. pastoris* GS115 (Figure 1). Empty plasmid without insert served as control and showed no band in agarose gel. In general, integrated plasmids attain low copy number than yeast replication plasmid. However, the integrated plasmid of the present study was highly stable for many generations under nonselective condition. The same plasmid was designed to integrate into the yeast genome at the* AOX* promoter, but the plasmid lacked* P. pastoris* specific autonomous replication sequence (PARS) which is crucial for the yeast genome [23]. Preliminary screening of* P. pastoris* transformants by PCR showed that a few recombinants harboured the pPICZ*α*B/*α*-amylase plasmid from GS115 strain which were subsequently used for protein expression.

### 3.3. Expression in Shake Flask

The effect of different media composition on the expression of recombinant *α*-amylase showed that YPTM (11.3 U mL^−1^) was found to be the best medium compared to BMMY and minimal media at 48 h (Figure 3(a)). The components of YPTM, peptone, and yeast extract is substantially rich in peptides, amino acids, vitamins, trace elements, and therefore had a prominent effect on the growth, biomass, and sufficient energy for protein synthesis during expression of recombinant proteins in* P. pastoris* [24]. Since methanol is the sole inducer for* P. pastoris* expression system, its concentration greatly affects the performance of heterologous protein production [25]. Concentration and induction of methanol plays a lead role in regulation of enzyme expression and methanol serves as an enhancer to increase the expression of recombinant enzyme. In the present study, induction of methanol at a concentration of 1% v/v resulted in high level of *α*-amylase SR74 expression compared to other concentrations (Figure 3(b)). This is in agreement with the fact that methanol-driven fermentation processes with* P. pastoris* typically consists of a biomass growth phase on glycerol and a protein production phase on methanol. Previously, an attempt was made to express the* G. stearothermophilus* derived *α*-amylase SR74 gene in bacterial expression system (*E. coli*) which resulted in an amylase production of 15.3 U mL^−1^. This amount was 9 folds higher than the *α*-amylase obtained from wild type bacterium (1.65 U mL^−1^). A maximum of up to 28.6 U mL^−1^ at 120 h after induction (Figure 3(c)) was achieved in* P. pastoris* transformants which was ~17 folds higher than that of wild type* G. stearothermophilus*. Overall, a two-fold increase in *α*-amylase productivity was achieved in* P. pastoris* expression system than the* E. coli*. Hence, it is clear that yeast expression system like* P. pastoris* would serve as a better expression system for enzymes like *α*-amylase SR74 in obtaining high cell density for the downstream process.

### 3.4. Purification of *α*-Amylase SR74

**Figure 2:**
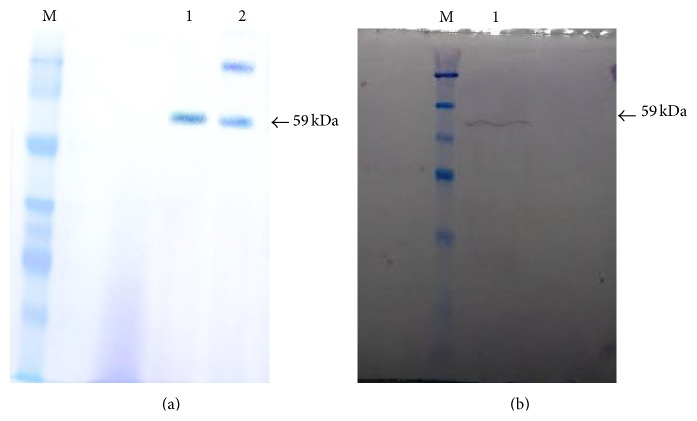
(a) SDS-PAGE (12%) analysis of purified *α*-amylase. M, unstained protein molecular weight marker; Lane 1, purified *α*-amylase SR74; Lane 2, crude extract of expressed *α*-amylase SR74. (b) Western blot analysis of recombinant *α*-amylase. M, unstained protein molecular weight marker; Lane 1, crude extract of *α*-amylase. Both arrows (←) indicate the molecular weight of *α*-amylase at 59 kDa.

**Figure 3:**
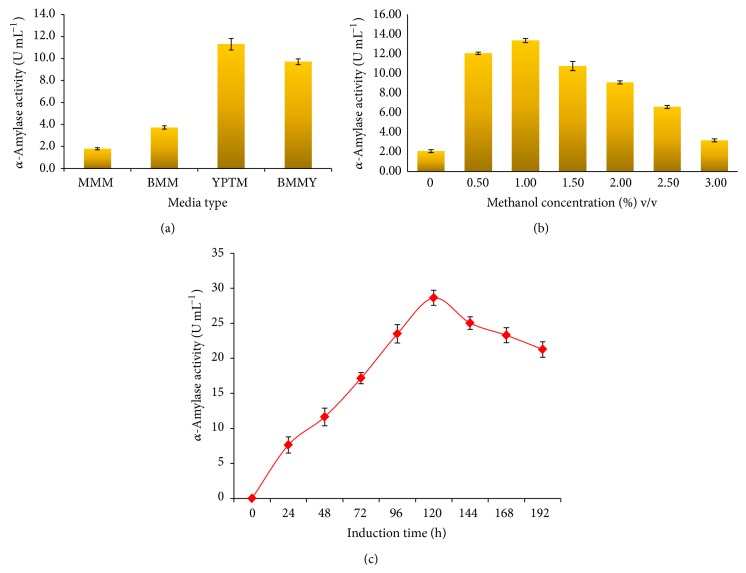
(a) Effect of various media on *α*-amylase SR74 activity at 48 h of cultivation of recombinant* P. pastoris *GS115 when induced with 0.5% (v/v) of methanol. Data are presented as ±SD of triplicates. (b) Effect of different methanol concentrations on *α*-amylase SR74 production by* P. pastoris *GS115 in 48 h cultivation in YPTM media. Data are presented as ±SD of triplicates. (c) Activity profile of recombinant *α*-amylase SR74 colony 21 of* P. pastoris.* Data are presented as ±SD of triplicates.

The single step purification of *α*-amylase by affinity chromatography resulted in a 52.6% of recovery with a purification fold of 1.9. The total activity for the purified *α*-amylase SR74 was about 267.2 U with total protein of 1.8 mg. However, the specific activity at the end of the purification step was found to be almost 151.8 U mg^−1^ compared to 79.4 U mg^−1^ of the crude (Table 2). It has been shown that the partially purified *α*-amylase obtained from* P. pastoris* by ammonium sulphate precipitation at 80% dialysis and membrane filtration was not very efficient which resulted in 11% yield of the desired enzyme. This relatively low level or recovery was because the majority of the obtained protein were lost or degraded during the precipitation steps [26]. However, techniques other than ammonium sulphate precipitation like gel filtration chromatography were found to be advantageous in obtaining the desired enzymes in substantial amounts. *α*-Amylase SR74 exhibited 1.9-fold increase with (52.6% yield) when compared to *α*-amylase produced by solid state fermentation which resulted in 1.2 folds (22% yield) productivity with a specific activity of 112 U mg^−1^ [27]. Use of Sephadex gel filtration for purification of *α*-amylase resulted in 1.5 fold increase in *α*-amylase and specific activity of 143 U mg^−1^ [27]. The present study utilized a single step affinity chromatography purification method (Figure 2(a)) which was found to be economical which reduces loss of protein/enzyme of interest while purification as well as resulted in a significant product yield for further characterization.

### 3.5. Western Blot Analysis

His-Tag monoclonal is a mouse monoclonal antibody (IgG) directly against the His-Tag sequence encoded by* P. pastoris* (pPICZ*α*B) expression vector. The sequence (6xHis) located near C-terminal after multiple cloning sites in pPICZ*α*B expression vector. The Anti-His (C-term) monoclonal antibody recognizes the sequence (6xHis). The secondary antibody Goat Anti-Mouse IgG AP conjugate bound to the primary antibody (IgG) and the signal was enhanced. Dark purple signal was observed at a molecular weight of 59 kDa (Figure 2(b)). The western blot analysis of the *α*-amylase SR74 fusion protein at 59 kDa showed the presence of (6xHis) the recombinant protein.

### 3.6. Characterization of *α*-Amylase

#### 3.6.1. Effect of pH

**Figure 4:**
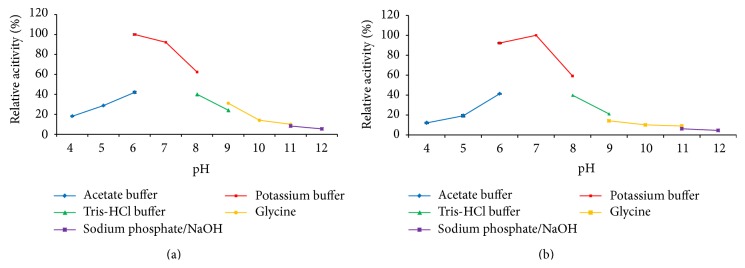
(a) The effect of pH on the activity of the purified recombinant *α*-amylase. The *α*-amylase was assayed at various pH conditions ranging from pH 4.0 to pH 12.0 and assayed by spectrophotometric means. Data are presented as ±SD of triplicates. (b) The effect of pH on the stability of purified recombinant *α*-amylase. The *α*-amylase was incubated at 60°C for 30 min at various pH conditions ranging from pH 4.0 to pH 12.0 and assayed by spectrophotometric means. Data are presented as ±SD of triplicates.

Optimum pH is defined as the pH at which an enzyme catalyzes a reaction at the maximum rate. The purified *α*-amylase SR74 in this study showed 50% more activity and was found to be highly active between pH 6.0 and 8.0. The optimum pH for *α*-amylase activity in this study was observed at pH 7.0 in 50 mM potassium phosphate buffer (Figure 4(a)). Moreover, it has been reported that phosphate buffer was the ideal pH compared to other buffers like acetate and tris, for extraction of *α*-amylases from malted finger millet [28]. Such enzymes obtained from neutral pH condition like potassium phosphate buffer are widely used in liquefaction of starch industry [29]. The recombinant *α*-amylase SR74 was stable by retaining more than 50% of the relative activity at pH ranging from 6.0 to 8.0 upon treatment in various buffers at 60°C for 30 min (Figure 4(b)). This study is one of first of its kind to reveal the optimum pH for* G. stearothermophilus* producing *α*-amylase SR74 (pH 7.0) as well as the wide range of pH stability (pH 6.0–8.0) thus making a potential advantage for commercialization.

#### 3.6.2. Effect of Temperature

**Figure 5:**
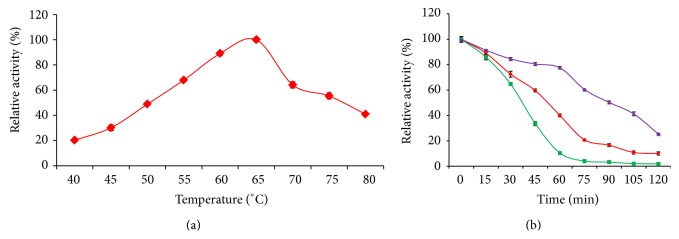
(a) The effect of temperature on the activity of the purified recombinant *α*-amylase. The *α*-amylase was assayed with starch as substrate in temperature ranging from 40°C to 80°C at pH 7.0 for 30 min. Data are presented as ±SD of triplicates. (b) Thermostability profile of purified recombinant *α*-amylase. The *α*-amylase was incubated at different temperature in potassium phosphate buffer (pH 7.0) and the activity was assayed at respective time interval. The symbols used are (▴) 60°C, (⧫) 65°C, and (■) 70°C. Data are presented as ±SD of triplicates.

The recombinant *α*-amylase SR74 was highly active at temperatures ranging from 55°C to 70°C with an optimum temperature at 65°C (Figure 5(a)). The *α*-amylase of other thermophilic strains like* Cryptococcus flavus*,* Lactobacillus manihotivorans*, and* Thermobifida fusca* were reported to be less thermostable with optimum activity at 50°C, 55°C, and 60°C, respectively. Interestingly, recombinant *α*-amylase SR74 of* G. stearothermophilus* was found to be thermophilic exhibiting optimum activity at 65°C [30–32]. Also the *α*-amylase SR74 retained its maximum activity (50–80%) at 60, 65, and 70°C with a half-life of 85 min at 60°C, 55 min at 65°C, and 40 min at 70°C, respectively (Figure 5(b)). Previously reported* Bacillus cereusα*-amylase retained a maximum activity of 75% with a half-life of 15 min [33], whereas* G. stearothermophilus α*-amylase SR74 was stable up to 40 min and declined drastically resulting in inactivation of enzyme. The thermostability of the enzyme reported in this study was higher. The high optimum and thermostability range allows this enzyme to be utilized in industrial applications, since it is time saving and cost saving and increased yield. The thermal properties exhibited by *α*-amylase SR74 of this study might suit the industrial needs and demands for application in gelatinization and liquefaction [6].

## 4. Conclusions

In conclusion, using yeast expression system like* P. pastoris*, high yield of *α*-amylase SR74 was achieved.* P. pastoris* are better than bacterial expression system like* E. coli*. The optimum temperature, pH, and high yield of* G. stearothermophilus* SR74 derived thermoactive and thermoactive *α*-amylase was reported. High success rate of recombinant protein of interest using* P. pastoris* expression system in a controlled environment provides a new hope for enzyme technologists whereas the high stability towards temperature, pH, and increased half-life proves its candidature as a new generation industrially important enzyme.

## Figures and Tables

**Table 1:** PCR conditions.

PCR conditions	Temperature (°C)	Time (min)	
Initial denaturation	94	4	

Denaturation	95	1	30 cycles
Annealing	65	1	
Extension	72	1	

Final extension	72	7	

Pause	4	0	

**Table 2:** Summary of the purification of His-tagged recombinant *α*-amylase.

Purification step	Total activity (U)	Total protein (mg)	Specific activity (U mg^−1^)	Recovery (%)	Purification (fold)
Crude	508	6.4	79.4	100	1
Ni-Sepharose	267.2	1.8	151.8	52.6	1.9

## References

[1] Ibrahim C. O. (2008). Development of applications of industrial enzymes from Malaysian indigenous microbial sources. *Bioresource Technology*.

[2] Janeček Š., Svensson B., MacGregor E. A. (2014). *α*-amylase: an enzyme specificity found in various families of glycoside hydrolases. *Cellular and Molecular Life Sciences*.

[3] Gupta R., Gigras P., Mohapatra H., Goswami V. K., Chauhan B. (2003). Microbial *α*-amylases: a biotechnological perspective. *Process Biochemistry*.

[4] Nielsen J. E., Borchert T. V. (2000). Protein engineering of bacterial alpha-amylases. *Biochimica et Biophysica Acta—Protein Structure and Molecular Enzymology*.

[5] van der Maarel M. J. E. C., van der Veen B., Uitdehaag J. C. M., Leemhuis H., Dijkhuizen L. (2002). Properties and applications of starch-converting enzymes of the *α*-amylase family. *Journal of Biotechnology*.

[6] Sivaramakrishnan S., Gangadharan D., Nampoothiri K. M., Soccol C. R., Pandey A. (2006). *α*-Amylases from microbial sources—an overview on recent developments. *Food Technology and Biotechnology*.

[7] Das S., Singh S., Sharma V., Soni M. L. (2011). Biotechnological applications of industrially important amylase enzyme. *International Journal of Pharma and Bio Sciences*.

[8] Divakaran D., Chandran A., Chandran R. P. (2011). Comparative study on production of *α*-amylase from *Bacillus licheniformis* strains. *Brazilian Journal of Microbiology*.

[9] Al-Qodah Z. (2006). Production and characterization of thermostable *α*-amylase by thermophilic *Geobacillus stearothermophilus*. *Biotechnology Journal*.

[10] Espargaró A., Villar-Piqué A., Sabaté R., Ventura S. (2012). Yeast prions form infectious amyloid inclusion bodies in bacteria. *Microbial Cell Factories*.

[11] Cereghino J. L., Cregg J. M. (2000). Heterologous protein expression in the methylotrophic yeast *Pichia pastoris*. *FEMS Microbiology Reviews*.

[12] Cregg J. M., Cereghino J. L., Shi J., Higgins D. R. (2000). Recombinant protein expression in *Pichia pastoris*. *Applied Biochemistry and Biotechnology Part B: Molecular Biotechnology*.

[13] Cereghino G. P. L., Cereghino J. L., Ilgen C., Cregg J. M. (2002). Production of recombinant proteins in fermenter cultures of the yeast *Pichia pastoris*. *Current Opinion in Biotechnology*.

[14] Marx H., Mecklenbräuker A., Gasser B., Sauer M., Mattanovich D. (2009). Directed gene copy number amplification in *Pichia pastoris* by vector integration into the ribosomal DNA locus. *FEMS Yeast Research*.

[15] Berrin J.-G., Williamson G., Puigserver A., Chaix J.-C., McLauchlan W. R., Juge N. (2000). High-level production of recombinant fungal endo-*β*-1,4-xylanase in the methylotrophic yeast *Pichia pastoris*. *Protein Expression and Purification*.

[16] Mattanovich D., Branduardi P., Dato L., Gasser B., Sauer M., Porro D. (2012). Recombinant protein production in yeasts. *Methods in Molecular Biology*.

[17] Ramchuran S. O., Mateus B., Holst O., Karlsson E. N. (2005). The methylotrophic yeast Pichia pastoris as a host for the expression and production of thermostable xylanase from the bacterium *Rhodothermus marinus*. *FEMS Yeast Research*.

[18] Bernfeld P. (1955). Amylases *α*- and *β*-methods. *Enzymology*.

[19] Bradford M. M. (1976). A rapid and sensitive method for the quantitation of microgram quantities of protein utilizing the principle of protein dye binding. *Analytical Biochemistry*.

[20] Laemmli U. K. (1970). Cleavage of structural proteins during the assembly of the head of bacteriophage T4. *Nature*.

[21] Weidner M., Taupp M., Hallam S. J. (2010). Expression of recombinant proteins in the methylotrophic yeast *Pichia pastoris*. *The Journal of Visualized Experiments*.

[22] Uehara H., Choi D. B., Park E. Y., Okabe M. (2000). Expression of mouse *α*-amylase gene in methylotrophic yeast Pichia pastoris. *Biotechnology and Bioprocess Engineering*.

[23] Latiffi A. A., Salleh A. B., Rahman R. N. Z. R. A., Nurbaya Oslan S., Basri M. (2013). Secretory expression of thermostable alkaline protease from *Bacillus stearothermophilus* FI by using native signal peptide and *α*-factor secretion signal in *Pichia pastoris*. *Genes and Genetic Systems*.

[24] Ling L. Y., Ithoi I., Yik F. M. (2010). Optimization for high-level expression in *Pichia pastoris* and purification of truncated and full length recombinant sag2 of *Toxoplasma gondii* for diagnostic use. *Southeast Asian Journal of Tropical Medicine and Public Health*.

[25] Wu J. M., Lin J. C., Chieng L. L., Lee C. K., Hsu T. A. (2003). Combined use of GAP and AOX1 promoter to enhance the expression of human granulocyte-macrophage colony-stimulating factor in *Pichia pastoris*. *Enzyme and Microbial Technology*.

[26] Karakaş B., Inan M., Certel M. (2010). Expression and characterization of *Bacillus subtilis* PY22 *α*-amylase in *Pichia pastoris*. *Journal of Molecular Catalysis B: Enzymatic*.

[27] Irshad M., Anwar Z., Gulfraz M., Butt H. I., Ejaz A., Nawaz H. (2012). Purification and characterization of *α*-amylase from Ganoderma tsuage growing in waste bread medium. *African Journal of Biotechnology*.

[28] Nirmala M., Muralikrishna G. (2003). Three *α*-amylases from malted finger millet (ragi, *Eleusine coracana*, Indaf-15)—purification and partial characterization. *Phytochemistry*.

[29] Fincan S. A., Enez B. (2014). Production, purification, and characterization of thermostable a-amylase from thermophilic *Geobacillus stearothermophilus*. *Starch/Staerke*.

[30] Aguilar G., Morlon-Guyot J., Trejo-Aguilar B., Guyot J. P. (2000). Purification and characterization of an extracellular *α*-amylase produced by *Lactobacillus manihotivorans* LMG 18010^T^, an amylolytic lactic acid bacterium. *Enzyme and Microbial Technology*.

[31] Wanderley K. J., Torres F. A. G., Moraes L. M. P., Ulhoa C. J. (2004). Biochemical characterization of *α*-amylase from the yeast *Cryptococcus flavus*. *FEMS Microbiology Letters*.

[32] Yang C.-H., Huang Y.-C., Chen C.-Y., Wen C.-Y. (2010). Expression of *Thermobifida fusca* thermostable raw starch digesting alpha-amylase in *Pichia pastoris* and its application in raw sago starch hydrolysis. *Journal of Industrial Microbiology and Biotechnology*.

[33] Mahdavi A., Sajedi R. H., Rassa M., Jafarian V. (2010). Characterization of an *α*-amylase with broad temperature activity from an acid-neutralizing *Bacillus cereus* strain. *Iranian Journal of Biotechnology*.

